# 

**Published:** 1892-07-16

**Authors:** 


**Notices of Births, Deaths, and Marriages,** are inserted in this column at a charge of 2s. 6d. for an announcement not exceeding four lines.

### NOTICE TO CORRESPONDENTS.

All MS., Letters, Books for Review, and other matters intended for the Editor should be addressed The Editor, The Lodge, Porchester Square, London, W.

The Editor cannot undertake to return rejected M.S., even when accompanied by stamped directed envelope.

**Notice to Advertisers and Subscribers.**

All Advertisements, Orders for Copies of The Hospital, and Business Communications should be addressed to The Publisher, *not to the Editor*, The Hospital, 140, Strand, W.C.

The Cost of Subscription, post free, is as follows:—Per week, 2½d.; per quarter, 2s. 6d.; per half-year, 5s.; per annum, 8s. 6d. Foreign:—Per annum, 10s. 8d.; payable, in all cases, in advance.

**"Burdett's Hospital Annual," 1891—1892.**

Third year of publication. 600 pp. Boards, gilt lettering. A most complete guide to British and Colonial Hospitals, Dispensaries, Nursing and Convalescent Institutions. and Asylums. Price 3s. 6d. Published by The Scientific Press (Limited), 140, Strand, W.C.

**NOTICE.—The Index for Vol. XI. (ending March, 1892) is on sale at the Office of this Journal, price 2½d., post free.**

### SCRAPS AND GLEANINGS.

DR. KOCH has been obliged to give up work for the present owing to ill health.

MR. D. P. CAMA has sent £105 to the Lord Mayor for the Mansion House poor-box.

THE sum of £2,127 13s. 6d. has been subscribed towards the Nuneaton Cottage Hospital.

THE Hospital Saturday Fund, Lincoln, amounted this year to £199 8s. 6d., and at Grantham to £81 9s. 3½d.

THE total amount for the Metropolitan Hospital Fund received at the Mansion House up to Friday last was £35,500.

THE Amalgamated Societies' Hospital Fund has given £76 to the Teddington and Hampton Wick Cottage Hospital.

A NEW pavilion hospital is to erected at Newcastle. The first wing or pavilion is to perpetuate the memory of the late Dr. Bruce.

THE Lady Mayoress's Rose Fête in aid of the Royal Hospital for Women and Children has realised £1,050, clear of expenses.

THE Bishop of Rochester distributed the prizes and medals to the students of the Medical School at Guy's Hospital on the 6th inst.

THE twentieth Congress of German Medical Men, which met last month at Leipzig, was attended by representatives of 149 societies.

THE Duke of Connaught has consented to become a patron of the Congress of the Sanitary Institution, to be held in Portsmouth, commencing September 12th.

DURING the past year 111 cases, inclusive of 26 cases of accident, were admitted to the Abingdon Cottage Hospital. The erection of a mortuary at a cost of £64 was found essential.

THE late Mr. R. A. Newbon has bequeathed £15,000, less duty, to endow a ward of the Great Northern Central Hospital, Holloway Road, which is to be named the Newbon Ward.

AT a special meeting of the Court of Governors of the Sussex County Hospital, Brighton, it was decided to erect a new special building for providing additional accommodation for women.

AFTER much delay and opposition the Burnley Board of Guardians have decided to build a new infirmary, and have accepted Messrs. A. and R. Parker's tender of £14,765 16s. for the work.

THE annual report of the Hospital for Diseases of the Throat, Golden Square, states that in 1891 a third ward was opened entirely for children. Already the seven cots in it had received 218 little patients.

THE Victoria Jubilee Hospital, at Swaffham, after having accomplished an excellent year's work shows a balance of £60, on its accounts, due to careful management and to the interest shown in the little institution in the neighbourhood.

THE Duke of York, Patron of the British and Foreign Sailors' Society, sent, before starting for his cruise in the *Melampus*, £20 towards the Albert Victor Sailors' Rest, which is to be erected in the West India Dock Road.

THE meeting held at the Mansion House in aid of the Children's Country Holidays Fund has resulted in the sum of £130 being handed over to Mr. Lyttelton. This sum will give a fortnight's holiday to about three hundred poor children.

THE Duchess of Bedford presented the prizes to the successful students of the London School of Medicine. During the year one of the students, Miss Porce, has gained the gold medal in obstetrics at the London University, and Miss Sturge has taken honours in Medicine.

THE British Medical Association is to celebrate its eightieth annual meeting at Nottingham on July 26th and three following days. The gathering promises to be a very large one this year, and will include many distinguished foreign professors of medical science.

MR. J. A. SMITH, of Northampton, was found dead by the roadside near Grafton Regis. He was one of the most successful cyclists in the Midland Counties, and had won between 150 and 200 prizes during the past twenty years. It is believed that death was due to over exertion.

A PERFORMANCE in aid of the Italian Hospital is to be given by Mr. Imre Kiralfy at Olympia on Wednesday, 20th inst., that being the birthday of the Italian Queen. The entertainment will be given with the support of the King and Queen of Italy, their Majesties being directly represented by the Italian Ambassador.

THE Council of the Middlesex Hospital has always given considerable attention to the protection of the buildings from fire, and has just purchased some additional hand fire engines and apparatus from Messrs. Merryweather, to supplement the high-pressure fire-mains fitted up by the same firm some years since.

DURING the past month the National Society for the Prevention of Cruelty to Children investigated 851 complaints of ill-treatment and neglect, affecting the welfare of 2,026 children. In 543 cases warnings were given, and in 88 the offences were so gross as to necessitate prosecution. The remainder were dealt with in other ways.

THE wife of a well-known Bradford cyclist recently met with a shocking death at Ben Rhydding, near Inkley. As they were passing a wagon the back wheel of the tricycle skidded, and both riders were thrown under the horses' feet. The machine was doubled up and the lady was killed, but her husband escaped with comparatively slight injuries.

AT a special general meeting of the Derbyshire Royal Infirmary it was decided to accept Messrs. Slater's, of London, tender for the engineering work to be done, amounting to £1?,094. The tender includes the whole of the hot water supply, the heating apparatus of all the rooms, the cold water supply, the boilers, a water softening apparatus and destructor, &c.

SOME Yorkshire ladies recently provided the patients (numbering 111) of the National Hospital for Consumption, Ventnor, with a most enjoyable Yorkshire feast of strawberries and cream, in commemoration of their brother's wedding, which took place the same day. A border of ivy flowers garlanded each table, and pot and cut flowers were placed about in profusion. The Town Band played several selections during tea.

### THE STUDENT OF MEDICINE.

[All communications for this Supplement should be addressed to THE EDITOR of THE HOSPITAL, at 140, Strand, with the words, "Students' Column," written in the left hand top corner of the envelope.]

### APPOINTMENTS.

Barratt, J. O. W., M.D.Lond., F.R.C.S., appointed Medica Officer at the Infirmary of the Parish of Birmingham. Betts, F. Bernard, M.R.C.S., L.R.C.P., appointed Resident Medical Officer to Queen Charlotte's Lying-in Hospital, Marylebone Road, N.W. Bolus, H. B., M.B., B.C.Cantab., appointed Obstetric Resident at Guy's Hospital. Carter, A. H., M.D.Lond., M.R.C.S., appointed Medical Officer at the Infirmary of the Parish of Birmingham. Close, John Borrill, M.B.Durh., L.R.C.P.Lond., M.R.C.S., appointed Honorary Surgeon to the Hull Orthopædic Hospital. Collins, Richard Hawtrey, M.R.C.S.Eng., L.R.C.P.Lond., appointed Senior House Surgeon to the Poplar Hospital for Accidents. Landon, E. E. B., M.R.C.S., L.R.C.P., appointed Obstetric Resident at Guy's Hospital. Lockwood, C. B., F.R.C.S.Eng., appointed Assistant Surgeon to St. Bartholomew's Hospital. Lowson, David, M.D.Aberd., L.R.C.S.Edin., appointed Honorary Surgeon to the Hull Orthopædic Hospital. Moritz, S., M.D., M.R.C.P.Lond., appointed Honorary Physician to the Manchester Hospital for Consumption and Diseases of the Throat and Chest. Nelson, Thomas, M.D., appointed Ingleby Examiner, Queen's College, Birmingham. Passmore, W. Edwin, L.S.A., appointed Assistant Medical Officer to the Infirmary of the Wandsworth Clapham Union. Radford, Willian J., L.R.C.P.Lond., M.R.C.S.Eng., appointed Assistant House Surgeon to the Poplar Hospital for Accidents, E. Savege, James, M.D.Aberd., appointed Honorary Anæsthetist to the Hull Orthopædic Hospital. Short, T. S., M.D.Lond., M.R.C.S., appointed Medical Officer at the Infirmary of the Parish of Birmingham. Sichel, G. M.R.C.S., L.R.C.P., appointed Obstetric Resident at Guy's Hospital. Tosswill, J. Cecil, M.B., C.M.Edin., appointed Visiting House Surgeon to the Stockport Infirmary.

### VACANCIES.

The following vacancies are announced this week, the amount of salary in each case being given after the name of the institution:

### Resident Medical Officers.

Borough Hospital, Birkenhead, Junior House Surgeon, doubly qualified —Salary, £60 per annum, with board, &c.
Evelina Hospital for Sick Children, Southwark Bridge Road, S.E., Junior Resident Medical Officer—Salary, £50 per annum.
Great Northern Central Hospital, Holloway Road, N., House Physician —Salary, £60 per annum, with board, &c.
Liverpool Northern Hospital, Assistant House Surgeon, doubly qualified —Salary, £70 per annum, with board, &c.
St. Luke's Hospital, Resident Clinical Assistant, appointment for six months—Board, &c.
Sheffield General Infirmary, Assistant House Surgeon, doubly qualified —Salary, £80 per annum, with board, &c.
Staffordshire General Infirmary, Stafford, Assistant House Surgeon—Board, &c.
Wonford House Hospital for the Insane, near Exeter, Assistant Medical Officer, doubly qualified, and not more than 28 years of age—Salary, £150 per annum, with board, &c.

### Non-Resident Medical Officers.

McGill University, Montreal, Canada, Professor of Pathology for the Faculties of Medicine and Comparative Medicine—Salary, £400 per annum.
Queen's Hospital, Birmingham, Physician for Out-patients and Pathologist, appointment for three years—Honorarium, £75 per annum.
University College Hospital. Assistant Opthalmic Surgeon.
Worcester Amalgamated Friendly Societies Medical Association, Assistant Medical Officer—Salary, £140 per annum, with a portion of midwifery fees, and £20 per annum for cab hire.

### PASS LISTS.

### University of Oxford.

SECOND M.B. EXAMINATION.

The following have passed: A. Adams, Exeter; A. Boyd, Christ Church; J. F. Broadbent, Hertford; H. R. Brown, Keble; J. Brungate, Christ Church; A. Butler-Harris, Trinity; A. Campbell, Hertford; E. Cartwright, Exeter; J. Earle, non-collegiate; R. Ellis, Magdalen; G. J. Maurice, Keble; M. Pembury, Christ Church; F. Woollacott, Queen's.

FIRST M.B. EXAMINATION.

The following have passed:
ANATOMY AND PHYSIOLOGY.—J. Buller, Corpus Christi; E. Collis, Keble; A. Cooke, New; R. Crawfurd, New; W. L. Fry, St. John's; E. Latham, Balliol; W. Robinson, non-collegiate; E. Savage, Christ Church; W. Stone, Balliol.
MATERIA MEDICA.—H. Barlow, Trinity; R. Bremridge, Magdalen; H. Cooper, Queen's; P. Dear, non-collegiate; T. Fisher, Brasenose; F.

### THE STUDENT OF MEDICINE—(*continued*).

Freemantle, Balliol; M. Gordon, Keble; E. Hopkinson, Trinity; R. Howard, Trinity; G. Maurice, Keble; R. Moon, New; E. Palin, Christ Church; L. Panting, Balliol; C. Reynolds, Keble; H. Sanguinetti, Balliol; A. Still, Christ Church; H. Vernon, Merton; W. Walters, Brasenose; R. Whittington, Merton.

ORGANIC CHEMISTRY.—R. Crawfurd, New; H. Foot, Lincoln; F. Freemantle, Balliol; M. Gordon, Keble; A. Gray, Magdalen; J. Hindley, Christ Church; R. Howard, Trinity; E. Palin, Christ Church; A. Parker, Magdalen; A. Partridge, Pembroke; C. Reynolds, Keble; W. Robinson, non-collegiate; H. Sanguinetti, Balliol; A. Still, Christ Church; D. Todd, Lincoln; W. Walters, Brasenose; T. Whigham, New; R. Whittington, Merton; A. Stewart, New; D. Stone, Balliol; R. Moon, New.

### Victoria University.

ENTRANCE EXAMINATION IN ARTS, FACULTY OF MEDICINE.

FIRST DIVISION.—T. F. Bamford, W. H. Bateman, H. A. Close, H. M. Cockcroft, H. Davies, J. Goodall, C. H. Moorhouse, J. E. H. Scott, R. B. Scott, R. Sutherland.

SECOND DIVISION.—F. H. Allen, G. L. Baker, T. T. Bark, T. Bayne, H. N. Bridge, A. G. S. Broughton, J. J. Butterworth, C. E. Craven, E. Deane, L. O. Delecourt, B. Gowing, A. S. Griffith, J. I. Halstead, W. D. Hamer, E. Hutton, H. Marriott, J. Milne, F. Morrisy, A. E. Normington, S. Pritchard, S. T. Rowling, W. Sankey, J. H. Willett, D. S. Wylie.

FIRST M.B. EXAMINATION. PART I.

CHEMISTRY AND PHYSICS.—E. L. Anderson, University College; G. Ashton, Owens College; J. T. Auld, Owens College; F. J. Batten, Owens College; E. Bennett, Owens College; J. W. Bennett, Owens College; R. Bleasdale, Owens College; A. W. Bollans, Yorkshire College; H. M. Brown, Owens College; J. B. Clarke, Owens College; H. M. Crake, University College; H. R. Cross, Yorkshire College; W. Cunliffe, Yorkshire College; F. Darlow, Yorkshire College; H. M. Henderson, University College; V. R. Hendry, University College; F. B. Holmes, Owens College; E. Ladyman, Owens College; A. T. Lakin, Owens College; J. R. Lambert, Yorkshire College; E. E. Laslett, University College; A. Lomas, Owens College; F. E. Marshall, University College; F. W. Morris, University College; N. Neild, Owens College; T. O'Neill, Owens College; G. C. Phipps, Owens College; A. M'L. Pilcher, Owens College; F. S. Pitt-Taylor, University College; A. H. Priestley, Owens College; A. L. Rhind, Owens College; J. S. Ross, Owens College; C. R. Schofield, Owens College; A. T. Sissons, Owens College; J. E. Smith; University College; H. H. Smith, Owens College; H. Stansfield, Yorkshire College; F. E. Taylor, Yorkshire College; H. Thorp, Owens College; W. C. Varley, Owens College; J. V. Watson, Owens College; A. Wightwick, Owens College; R. H. Wilshaw, Owens College; A. A. Wood, University College; J. Wood, Owens College; H. C. Woodhouse, Owens College.

DOCTOR OF MEDICINE.—J. M. H. Martin, University College.

BACHELOR OF MEDICINE AND OF SURGERY.—First-class honours: J. W. Crawshaw, Owens College; J. H. Taylor, Owens College. Second-class honours: A. E. Ash, Owens College; E. C. McCarthy, Owens College; R. W. Marsden, Owens College; R. T. Turner, Owens College. Past list: A. Bicknell, University College; A. J. Edwards, Owens College; J. H. Lightbody, University College; C. E. M. Lowe, Owens College; C. B. Taylor, Owens College.

### Conjoint Examinations (Colleges of Physicians and Surgeons) in Ireland.

FIRST PROFESSIONAL EXAMINATION.—The following candidates have passed: J. C. Baskin, J. W. Benson, W. J. Beveridge, G. E. Caithness, D. F. Clarke, H. F. Conyngham, G. Corcoran, W. M. Cummins, G. G. Delap, J. C. Edge, E. A. Fenton, T. Fitzgerald, R. Glynn, R. M. Hamilton, P. P. Harold-Barry, W. J. Healey, C. E. Hodson, C. R. Horrell, W. E. Joliffe, W. M. Jones, C. A. Kenny, C. B. Martin, H. B. S. Montgomery, J. Morrissey, J. A. M'Munn, M, O'Brien, W. H. Odlum, J. J. O'Reilly, T. J. Perkins, T. J. Potter. F. J. Purcell, A. S. Sampey, R. W. Scully, J. N. Shee, E. W. Siberry, Miss L. F. S. Strangman, Miss M. S. P. Strangman, R. H. Walpole, F. C. Wright, A. H. O. Young.

### Dublin.

At the Summer Commencements, held in Trinity Term, on Thursday, June 23rd, 1892, in the Examination Hall of Trinity College, the following degrees in Medicine, Surgery, and Midwifery were conferred by the University Caput in the presence of the Senate:—

BACCALAUREI IN MEDICINA, IN CHIRURGIA, ET IN ARTE OBSTETRICIA.—J. B. Anderson. W. Bleazby, R. J. Coulter, J. A. Cree, C. C. Deane, J. Elliott, G. G. Fannin, M. L. Griffin, R. H. Kennan, S. Matthews, F. W. Staunton, C. E. Stokes, J. Thompson, A. E. Wales, C. E. W. Wilmot.

DOCTORES IN MEDICINA.—T. Bell, J. Blood, R. A. C. Burnes, R. L. Joynt, R. F. M'Craith, T. Myles, B. M'Carthy, R. W. H. Jackson, R. D. Purefoy, A. E. Wales. (*In absentia*) H. H. Fleming.

---

### ATHLETICS.—Lawn Tennis.

INTER-HOSPITAL CHALLENGE CUP.—The annual competition for the Inter-Hospital Challenge Cup was concluded at Richmond on Friday Guy's Hospital, the holders, proving successful over St. Thomas's Hospital in the final round by five matches to one. Results:—Singles: G. Webb (Guy's) beat C. A. N. Knox (St. Thomas's) (6—4, 6—4, 6—2), T. Davies (St. Thomas's) beat E. Reid (Guy's) (6—2, 2—6, 6—1, 6—4), W. J. Hancock (Guy's) beat G. W. Bird (St. Thomas's) (6—2, 6—2, 6—1). Doubles: E. D. Hancock and Peake (Guy's) beat A. E. Thorpe and A. Hood (St. Thomas's) (6—3, 6—2, 6—3), W. J. Hancock and E. Reid (Guy's) beat G. W. H. Bird and C. A. N. Knox (St. Thomas's) (6—2, 8—6, 6—1, 4—6), Webb and Coulson (Guy's) beat Davies and Ransome (St. Thomas's) (4—6, 6—1, 7—5, 12—12, retired).

